# Performance of large language models in prescription review: a comparative study

**DOI:** 10.3389/fmed.2026.1939786

**Published:** 2026-10-01

**Authors:** Can Huang, Yanfang Sun, Wei Liu

**Affiliations:** Beijing Youan Hospital, Capital Medical University, Beijing, China

**Keywords:** large language models, medication safety, pharmaceutical care, pharmacist skills, prescription review

## Abstract

**Objective:**

Large language models (LLMs) have emerged as a novel technical solution for intelligent prescription review. Nevertheless, comparative analyses of their performance remain limited. This study comprehensively evaluates five mainstream LLMs (ChatGPT, DeepSeek, Kimi, Qwen and Doubao) in prescription review, aiming to furnish data-based references for model selection, optimization and clinical deployment of intelligent prescription review systems.

**Methods:**

This study employed a cross-sectional comparative design. The evaluation dataset was derived from the prescription review question bank of the Chinese Pharmacist Skills Competition. In total, 100 prescriptions from 10 clinical specialties were randomly sampled, encompassing 270 standardized medication assessment items. Official standard answers served as the gold standard. Quantitative comparisons were performed on overall detection rate, question type identification and adaptability across clinical specialties. Prescription-level repeated-measures analysis of variance (ANOVA) and Holm-adjusted pairwise paired *t*-tests were used to examine inter-model statistical differences.

**Results:**

The five models yielded item-level prescription detection rate ranging from 45.19% to 70.37% across 270 standardized medication assessment items. Their performance ranked from highest to lowest as follows: Kimi, DeepSeek, Qwen, Doubao and ChatGPT. Overall, the four Chinese domestic models included in this study outperformed the single tested international model (ChatGPT) in this China-focused prescription review task. Repeated-measures ANOVA confirmed statistically significant within-prescription differences in overall performance (F = 22.46, *P* < 0.001). While all models performed well at identifying inappropriate dosage forms and administration routes, they were less effective in detecting issues involving absent allergy test documentation and flawed result interpretation. Prescription reviews for the respiratory department yielded the best results, whereas detection rate was substantially lower for specialized clinical specialties such as oncology, pediatrics and pain management. Even the best-performing model presented a high false-negative rate and cannot yet satisfy the strict reliability criteria for clinical application in prescription review.

**Conclusion:**

Performance varied markedly across the five LLMs in prescription review. Although these models can effectively identify routine and straightforward medication issues, they demonstrate prominent deficiencies in assessing complex prescriptions from specialist clinical specialties and detecting hidden medication risks. Currently, LLMs are only suitable for preliminary auxiliary screening and cannot substitute dedicated prescription review software or manual pharmacist evaluation.

## Introduction

Prescription review constitutes a core part of pharmaceutical care and an essential safeguard for patient medication safety and the reduction of adverse drug events (ADEs) (1–3). Research demonstrates that irrational prescribing and inappropriate medication use are leading contributors to preventable ADEs. Rigorous and standardized prescription review can reduce medication errors and ADEs by over 50% (4–6). China's Standards for Prescription Review in Medical Institutions mandates mandatory review and approval of all prescriptions before dispensing. The accuracy, comprehensiveness and timeliness of prescription review are critical determinants of medical quality and patient safety (7).

Traditional manual prescription review in clinical settings is highly dependent on pharmacists' professional proficiency, clinical experience and working status. Daily workloads ranging from hundreds to thousands of prescriptions inevitably lead to low efficiency, inconsistent enforcement of review standards and variable performance in specialized medication review. The workforce shortage of professional pharmacists, especially in primary care settings and specialist outpatient services, further impedes quality improvement in prescription review (8, 9).

Driven by advances in artificial intelligence (AI) and natural language processing (NLP), large language models (LLMs)-which feature robust knowledge acquisition, contextual comprehension and logical reasoning-have emerged as a novel approach to the intelligent transformation of prescription review (10, 11). Leading LLMs including ChatGPT and DeepSeek have exhibited considerable potential across diverse medical applications, such as medical literature analysis, electronic medical record structuring, medication counseling and patient health education (12–15). Unlike conventional rule-based prescription review software, LLMs deliver superior text understanding and flexible reasoning, enabling them to better cope with the complex wording and individualized features of clinical prescriptions. As such, they have become a key research focus in intelligent pharmaceutical care (16).

However, most existing works are limited to exploratory applications. For the highly professional and rigorous task of prescription review, systematic quantitative evaluation using standardized frameworks remains scarce. The real-world performance of general-purpose LLMs, including their error detection rate, sensitivity, adaptability to various question types and cross-department applicability, has not been objectively and comprehensively verified.

To evaluate the practical performance of LLMs in prescription review, this study employed test items sourced from the Chinese Pharmacist Skills Competition as standardized evaluation materials. Developed by national clinical pharmacy experts, these items target core competencies for prescription review, encompass prevalent medication issues across clinical specialties, and adhere to uniform scoring criteria with high professional rigor, which fully reflects the practical demands of clinical prescription review. A total of 100 prescriptions from 10 clinical specialties were randomly sampled from the question bank for comparative testing of mainstream LLMs.

Using consistent scoring protocols, this study identifies the strengths, limitations and practical boundaries of general-purpose LLMs in prescription review. The results are intended to inform model selection for hospital-based intelligent prescription review systems, guide the professional optimization of LLMs for pharmacy practice, accelerate the safe, compliant and efficient deployment of AI in pharmaceutical care, and enhance prescription review quality and rational drug use.

## Materials and methods

### Test sample source and construction

The Chinese Pharmacist Skills Competition is a national pharmacy skills contest jointly hosted by the Chinese Aging Well Association, the Zhongguancun Medical Health Science and Technology Innovation Center and other organizations. Initiated in 2021 and held on an annual basis, it is one of China's most influential and professionally recognized pharmacy competitions with extensive coverage. The competition's prescription review question bank is developed by clinical pharmacy experts using real clinical cases and up-to-date medication guidelines. It spans multiple clinical specialties and disease entities, focusing on core pharmacy competencies such as prescription rationality, medication suitability, drug interactions and contraindications. The question bank features high authority, standardization and clinical practicality. All test specimens used in this study were obtained from this official resource.

Random sampling was applied to select prescriptions from 10 clinical specialties: respiratory medicine, gastroenterology, neurology, cardiovascular medicine, hematology, endocrinology, orthopedics, pediatrics, oncology, and pain management. Ten prescriptions were collected from each specialty, resulting in a total of 100 prescriptions. In the original competition question bank, each prescription had been labeled by clinical pharmacy experts according to its primary diagnosis and therapeutic area. These labels were used to assign each prescription to a clinical specialty. After secondary review by clinical pharmacy specialists, a total of 270 standardized medication assessment items were identified within the selected prescriptions.

The average number of assessment items per prescription was 2.7. The item count per clinical specialty varied from 22 to 32; pediatrics had the largest number (32), while neurology had the smallest (22). The 270 items were classified into eight types of medication errors (Table 1). All items were text-only, without any images, tables or other multimedia elements.

**Table 1:** Distribution of prescription medication question types.

Question Type	Total Score
Incorrect Usage and Dosage	94
Presence of Medication Contraindications	44
Medication Inconsistent with Diagnosis	41
Presence of Drug Interactions	33
Inappropriate Solvent Selection	26
Inappropriate Selection of Dosage Form and Administration Route	18
Presence of Duplicate Drug Administration	12
No Indication of Allergy Test and Result Assessment	2
Total	270

Each of the 100 prescriptions was independently reviewed by two clinical pharmacy specialists. All medication-related problems identified in the official answer key were extracted and operationalized as predefined assessment items. A total of 270 predefined medication problems were identified. These items were not separate examination questions; they were the complete set of scoring points embedded within the 100 prescription cases. The models were instructed to identify all medication-related problems in each prescription. Therefore, the primary outcome was the item-level detection rate (sensitivity), defined as the number of predefined medication problems correctly detected divided by 270, multiplied by 100%.

### Evaluation models

This study evaluated five mainstream LLMs widely available and applied in China. All models were accessed through official public web interfaces instead of application programming interfaces (APIs). No external tools, plug-ins or auxiliary software were adopted throughout the evaluation process. Internet access was enabled for all LLMs to support real-time information retrieval. All models ran with default parameter configurations for response generation to guarantee consistent experimental conditions across tests.

To ensure the reproducibility of the experiment, we explicitly specified the web version numbers of all models, which remained unchanged throughout the entire testing period (April 8–10, 2026) as verified by official platform release logs. Additionally, consistent language and regional settings were configured for all models: the interface language was set to Simplified Chinese, and the regional setting was set to Mainland China, to prioritize Chinese medical corpora, local clinical guidelines, and domestic pharmaceutical regulations in model responses. The evaluated models and their corresponding web versions are as follows:

- ChatGPT: Developed by OpenAI; official web version: gpt-4o-mini
- DeepSeek: Developed by DeepSeek Inc.; official web version: deepseek-chat
- Kimi: Developed by Moonshot AI; official web version: kimi-k2
- Qwen: Independently developed by Alibaba Group; official web version: qwen3.5-plus
- Doubao: Developed by ByteDance; official web version: doubao-seed-2-0-pro

### Testing procedure and evaluation metrics

Data collection took place from April 8 to 10, 2026. A standardized testing protocol was followed to guarantee objectivity and reproducibility.

Standardized prompt (Chinese original):

“你是一名临床药师，请依据《医疗机构处方审核规范》审核以下处方。患者信息：{性别}，{年龄}；临床诊断：{诊断}；注释：{体重、过敏史等}。处方：{药品名称、规格、用法用量}。请逐条列出该处方中所有不合理的用药问题，并简要说明理由。若未发现问题，请回答‘未发现明显不合理用药’。”

English translation:

“You are a clinical pharmacist. Please review the following prescription according to the Standards for Prescription Review in Medical Institutions. Patient information: {sex}, {age}; Clinical diagnosis: {diagnosis}; Notes: {weight, allergy history, etc.}. Prescription: {drug name, strength, dosage and administration}. Please list all inappropriate medication problems in this prescription one by one and briefly explain the reason. If no problem is found, please answer ‘No obvious inappropriate medication identified’.”

Independent session testing with strict anti-interference procedures: Each prescription was tested in a separate blank session to prevent carry-over effects from historical context. To further eliminate potential interference from platform caches and residual session data, we implemented a multi-layer context clearing mechanism: All tests were conducted in the browser's incognito/private mode to prevent local cache retention; the browser was completely closed and restarted after completing tests for each model to clear all temporary data and session cookies; we logged out and re-logged into each model's official account before initiating a new batch of prescription tests; the session interface was verified to be completely blank with no historical conversation records before inputting each prescription.

#### Scoring rule

Each model response was independently mapped to the 270 predefined medication problems by two Supervising Pharmacists. For each predefined item, a score of 1 was assigned only when the model explicitly identified the target medication problem and provided a reason consistent with the official answer; otherwise, a score of 0 was assigned. Partially correct responses were scored 0 unless the target problem and its correct reason were both present. If a response contained multiple findings, each predefined item was scored independently. Additional incorrect findings not present in the predefined answer key were recorded but were not included in the primary outcome because the gold standard contained only predefined positive items.

#### Quality control

Two supervising pharmacists experienced in the Chinese Pharmacist Skills Competition independently documented the model outputs and graded the responses against the official answer key. Inter-rater reliability was assessed using Cohen's kappa based on the 270 paired item-level binary ratings. The initial independent scoring of all 270 items yielded a raw agreement rate of 97.04% and a Cohen's kappa coefficient of 0.938 (95% CI: 0.897–0.979), indicating almost perfect inter-rater reliability. Eight discrepant items were identified and resolved through a standardized two-step arbitration process: first through joint discussion between the two pharmacists with reference to the official answer key and the Standards for Prescription Review in Medical Institutions, and then through final adjudication by a senior clinical pharmacy researcher for any remaining disagreements.

#### Evaluation metrics

Primary outcome measures included total score (number of correctly detected medication problems) and detection rate (correct answers/total questions × 100%). Analyses were performed across overall performance, question categories and clinical specialties.

#### Repeated testing

Two replicate tests were administered 24 h apart to assess response stability and reliability. Each replicate used an independent session with no retention of prior data, following the same anti-interference procedures described above. The first round was used for the primary between-model analysis, whereas the second round was used solely for test-retest reliability assessment based on the intraclass correlation coefficient (ICC).

The detailed scoring results of each assessment item for all 5 models based on this rule are fully presented in the corresponding worksheets of the Supplementary File.

### Statistical analysis

All raw data were collated with Microsoft Excel 2021, and statistical analyses were conducted via SPSS 26.0. Counts, proportions and means were calculated for descriptive summaries. The primary unit of inferential analysis was the prescription. For each model, the prescription-level score was defined as the number of correctly identified assessment items in that prescription. Because all five models evaluated the same 100 prescriptions, model was treated as a within-prescription factor, and repeated-measures ANOVA was used to compare overall performance. Pairwise model comparisons were performed using paired *t*-tests with Holm adjustment for multiple comparisons. The first test round was used for the primary inferential analysis; the independent second test round was used only to calculate the ICC for test-retest reliability and was not pooled with the first round. Item-level detection rates were reported descriptively and were not treated as independent observations. All statistical tests were two-tailed, and *P* < 0.05 was considered statistically significant. ICC results were interpreted based on widely recognized clinical statistical criteria: poor agreement (<0.5), moderate agreement (0.5–0.75), good agreement (0.75–0.9), and excellent agreement (>0.9).

## Results

### Overall performance of each model

The item-level prescription detection rates of the five models differed significantly. Kimi ranked first with a detection rate of 70.37% (190/270), followed by DeepSeek with 64.81% (175/270), Qwen with 56.67% (153/270), Doubao with 52.22% (141/270), and ChatGPT lowest with 45.19% (122/270). The overall performance results are shown in Table 2 and Figure 1.

**Table 2:** Summary of overall prescription review performance of LLMs (detection rate = number of correctly identified medication assessment items/270 total assessment items × 100%).

Model	Total Score	Total Questions	Missed Items	Detection rate (%)	Rank
Kimi	190	270	80	70.37	1
DeepSeek	175	270	95	64.81	2
Qwen	153	270	117	56.67	3
Doubao	141	270	129	52.22	4
ChatGPT	122	270	148	45.19	5

**Figure 1:**
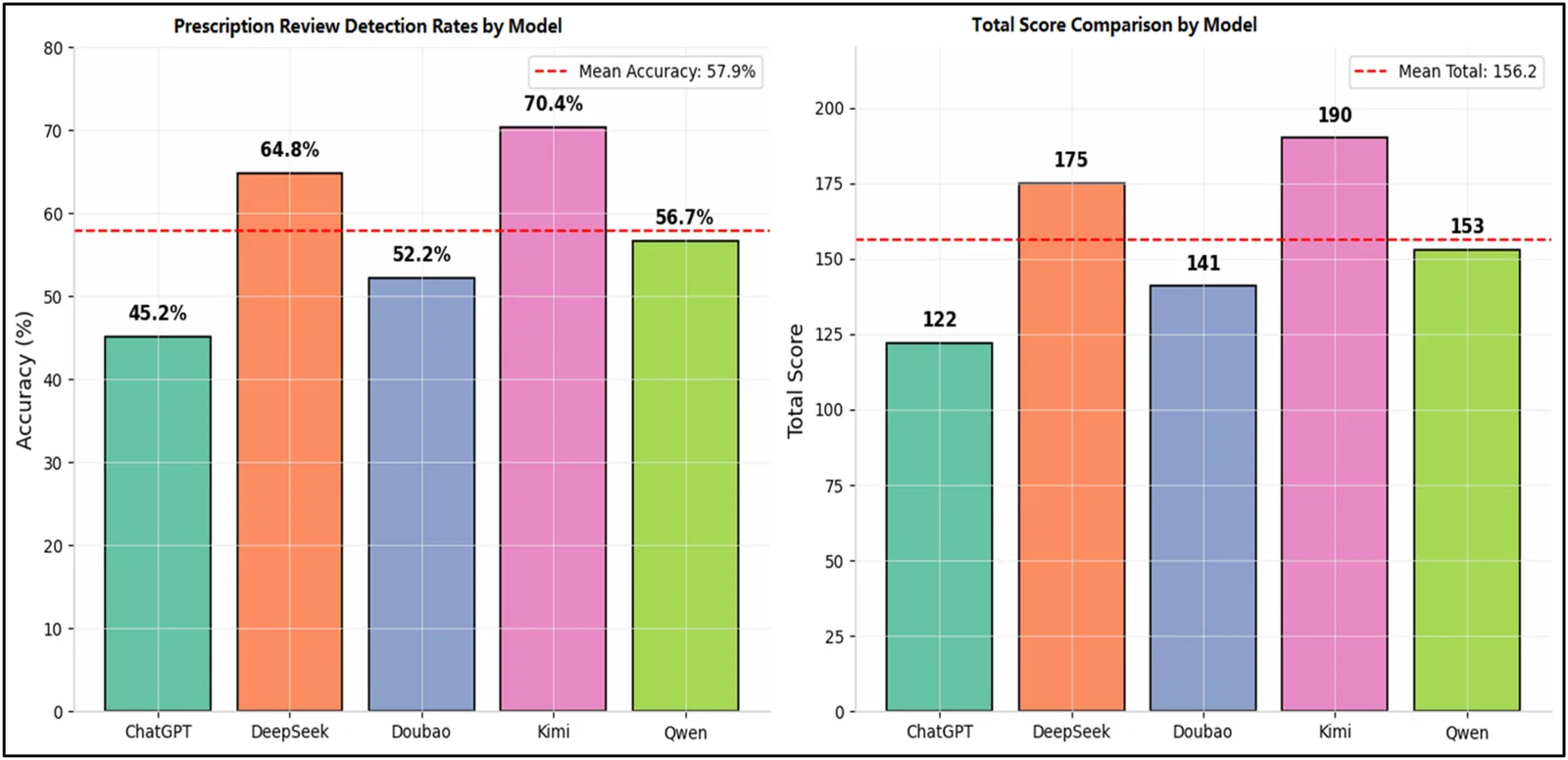
Comparison of prescription review performance among different LLMs.

Descriptive statistics of the average score per prescription (Figure 2) showed that Kimi had the highest mean score (1.90 ± 0.07), followed by DeepSeek (1.75 ± 0.08), and ChatGPT the lowest (1.22 ± 0.06).

**Figure 2:**
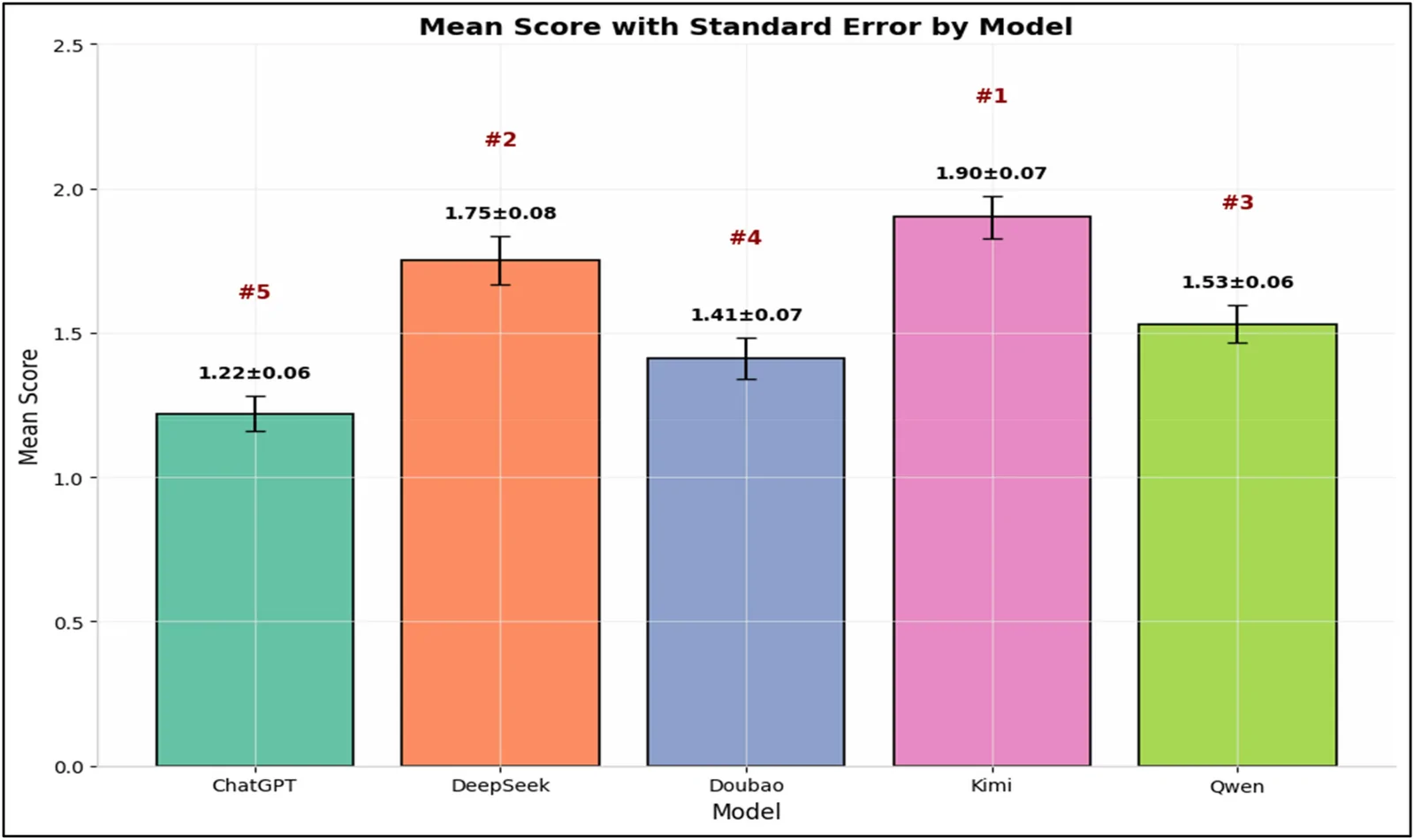
Average prescription review scores of each model.

A repeated-measures ANOVA based on per-prescription scores revealed significant differences in overall prescription review performance across the five LLMs (F = 22.46, *P* < 0.001). Holm-adjusted pairwise paired *t*-tests demonstrated that Kimi achieved a significantly higher score than Qwen, Doubao, and ChatGPT, while no significant difference was found between Kimi and DeepSeek. DeepSeek performed significantly better than Qwen, Doubao, and ChatGPT. For the two medium-performance models, Qwen and Doubao did not differ significantly; both models performed significantly better than ChatGPT.

### Test-retest reliability

ICC test-retest analysis revealed distinct response consistency levels among the five LLMs across two independent test rounds (Table 3). Kimi, ChatGPT and DeepSeek presented excellent response stability with ICC values greater than 0.90, among which Kimi obtained the highest ICC value of 0.949. This finding verified superior repeatability of the above three models in repeated prescription review tests. Qwen and Doubao attained good consistency, with ICC values ranging from 0.82 to 0.84; their stability was inferior to that of the top three models but still met the acceptable reliability standard. Meanwhile, Doubao had the largest mean response difference of +0.082. All ICC analyses yielded *P* values below 0.001, confirming statistically significant test-retest reliability for all included LLMs.

**Table 3:** ICC analysis.

Model	ICC	95% CI	Interpretation
Kimi	0.949	[0.925, 0.966]	Excellent
DeepSeek	0.909	[0.867, 0.938]	Excellent
Qwen	0.839	[0.769, 0.889]	Good
Doubao	0.818	[0.741, 0.875]	Good
ChatGPT	0.936	[0.876, 0.988]	Excellent

We analyzed the test-retest inconsistency patterns of Doubao and Qwen across question types and clinical specialties. For Doubao, highest inconsistencies occurred in drug interactions and diagnosis-medication mismatch. For Qwen, fluctuations were prominent in diagnosis mismatch and drug interactions. Both models showed high stability in rule-based categories such as dosage form/route errors. By clinical specialty, the greatest fluctuations were observed in oncology, pediatrics, and pain management-clinical specialties demanding complex, multi-guideline reasoning.

### Performance by question type

Substantial differences were observed across models in detecting distinct categories of prescription errors. The category of inappropriate dosage form and administration route had the highest overall average detection rate of 77.78%, and Kimi achieved the top performance (88.89%) in this regard. Medication contraindications and duplicate drug administration also had relatively high average detection rates, at 68.18% and 63.33%.

Conversely, failure to document allergy tests and interpret corresponding results was the most difficult item, with an average detection rate of just 10.00%. Kimi was the only model that correctly answered one of the two related questions (50%), while the other four models missed all relevant items. Medication inconsistent with diagnosis also showed a low average detection rate of 41.46%, with ChatGPT (19.51%) and Qwen (17.07%) demonstrating notably weak performance. Full results for the eight error categories are summarized in Table 4 and Figure 3.

**Table 4:** Detection rates of each model on different question types (%).

Question Type	Total	ChatGPT	DeepSeek	Doubao	Kimi	Qwen
Incorrect Usage and Dosage	94	44.68	78.72	59.57	74.47	63.83
Inappropriate Solvent Selection	26	57.69	46.15	26.92	53.85	38.46
No Indication of Allergy Test and Result Assessment	2	0.00	0.00	0.00	50.00	0.00
Inappropriate Selection of Dosage Form and Administration Route	18	72.22	83.33	77.78	88.89	66.67
Medication Inconsistent with Diagnosis	41	19.51	53.66	58.54	58.54	17.07
Presence of Drug Interactions	33	30.30	39.39	30.30	60.61	72.73
Presence of Medication Contraindications	44	61.36	75.00	52.27	81.82	70.45
Presence of Duplicate Drug Administration	12	58.33	50.00	58.33	75.00	75.00

**Figure 3:**
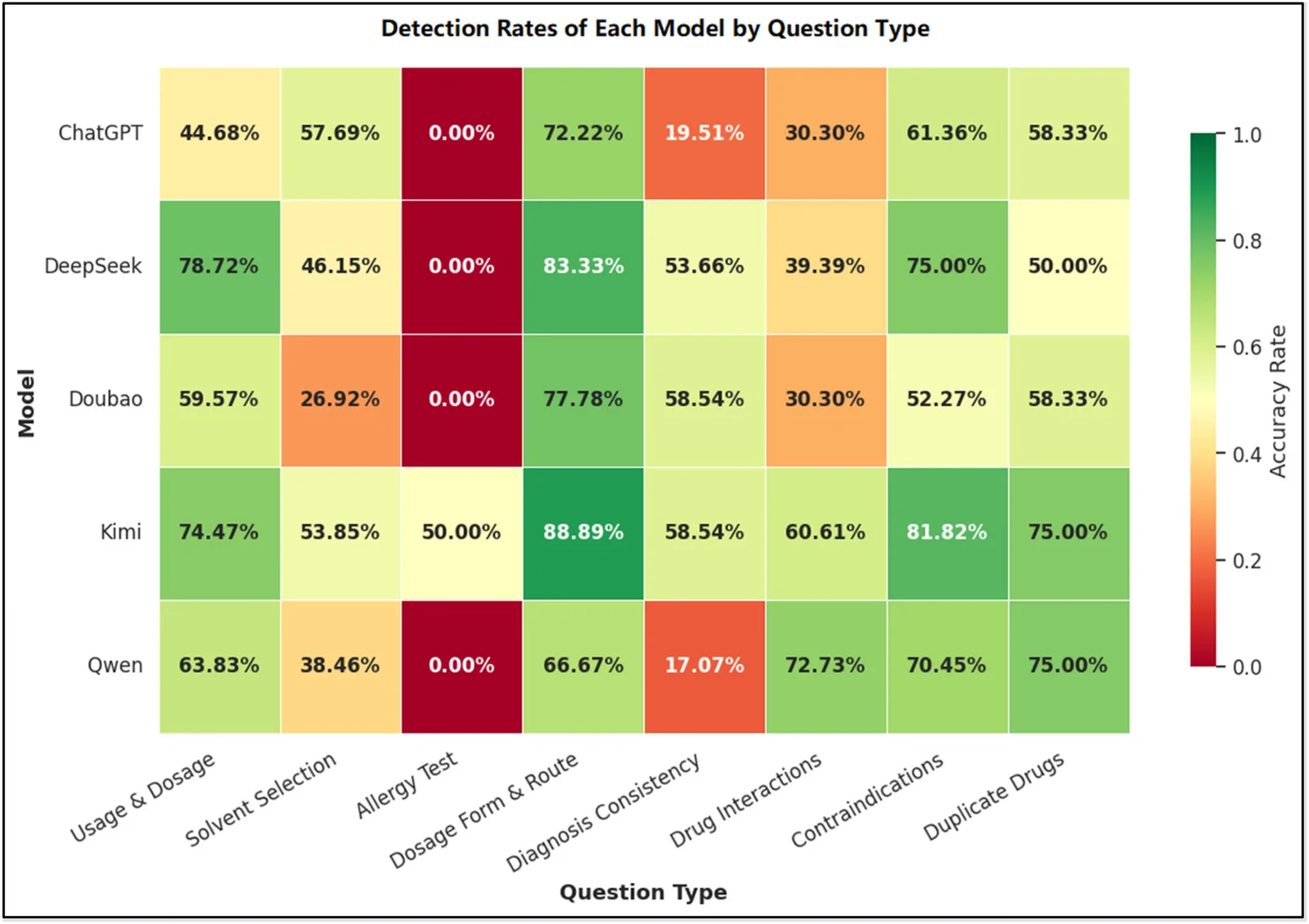
Heatmap of each model's ability to recognize eight types of prescription medication problems.

The performance disparity across question types stems from task nature. Rule-based tasks (e.g., dosage form/route identification) rely on explicit textual matching, aligning with LLMs' pattern-recognition strengths. In contrast, detecting missing allergy tests and diagnosis-medication mismatches demands implicit information extraction and cross-domain reasoning-integrating guidelines, indications, and patient factors.

### Performance by clinical specialty

Marked differences across clinical specialties were observed in model performance. Kimi maintained a detection rate exceeding 60% across nine clinical specialties, with oncology being the only exception at 57.14%. DeepSeek performed well in seven clinical specialties, showing a detection rate comparable to Kimi in respiratory medicine and neurology. ChatGPT achieved acceptable results only in respiratory medicine (74.07%) and neurology (63.64%), with notably low detection rates in orthopedics (32.14%) and pediatrics (34.38%).

Based on the overall average detection rate, prescriptions from respiratory medicine (74.07%) and neurology (68.18%) were easier to review. By contrast, oncology (47.14%) and pediatrics (50.63%) represented more difficult cases. This trend can be explained by the complexity of oncological treatment plans (e.g., chemotherapy and targeted therapy) and the strict regulations for pediatric prescriptions, including dose adjustment and age limitations. Detailed specialty-specific detection rates are shown in Table 5 and Figure 4.

**Table 5:** Detection rates of each model by clinical specialty (%).

Clinical specialty	Total Questions	ChatGPT	DeepSeek	Doubao	Kimi	Qwen
Respiratory Medicine	27	74.07	74.07	66.67	81.48	74.07
Gastroenterology	26	53.85	53.85	57.69	69.23	69.23
Neurology	22	63.64	63.64	54.55	90.91	68.18
Cardiovascular Medicine	27	44.44	70.37	51.85	70.37	66.67
Hematology	28	39.29	57.14	53.57	75.00	42.86
Endocrinology	25	44.00	76.00	60.00	68.00	68.00
Orthopedics	28	32.14	64.29	46.43	60.71	53.57
Pediatrics	32	34.38	53.13	46.88	68.75	50.00
Oncology	28	35.71	60.71	42.86	57.14	39.29
Pain Management	27	37.04	77.78	44.44	66.67	40.74

**Figure 4:**
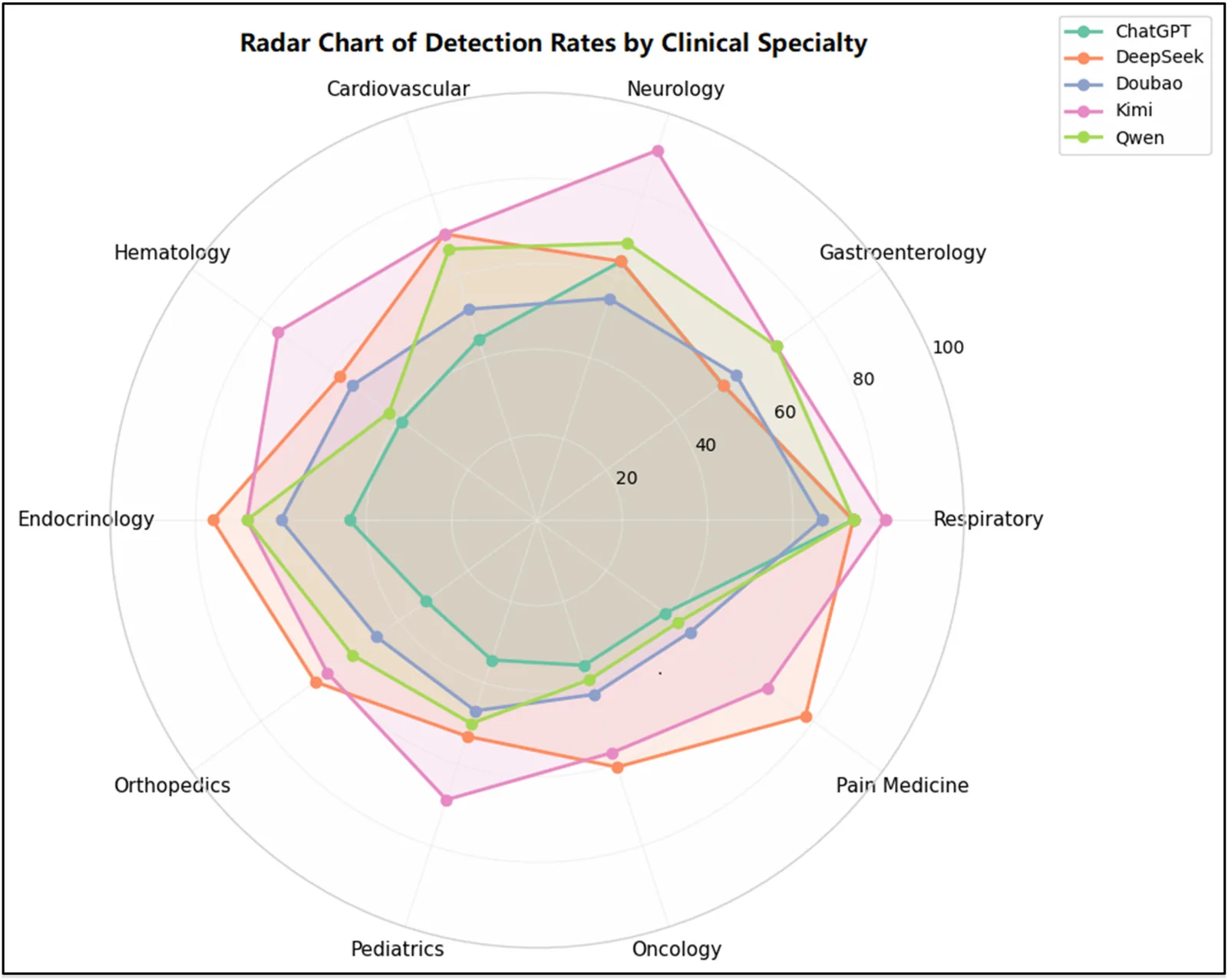
Radar chart of prescription detection rates by clinical specialty for each model.

The clinical specialties of respiratory medicine and neurology have standardised protocols and common diseases well-represented in training data, yielding higher detection rates. In contrast, oncology, paediatrics, and pain management involve complex, individualised regimens (chemotherapy, weight-based dosing) requiring nuanced judgment and specialised guidelines.

### Inter-model agreement and error pattern analysis

Across all 270 assessment items, the full inter-model agreement rate (all 5 models yielding identical correct/incorrect judgments) was 51.48%, indicating substantial heterogeneity in model performance across different error types. Agreement patterns varied markedly by error category, as summarized in Table 6.

**Table 6:** Inter-model full agreement rate by medication error type.

Error Type	Total Items	Items with full agreement across 5 models	Full agreement rate (%)	Dominant error pattern
Inappropriate selection of dosage form and administration route	18	14	77.78	High consistency; rule-based explicit errors
Presence of duplicate drug administration	12	8	66.67	High consistency; explicit pharmacological matching
Incorrect usage and dosage	94	58	61.70	Moderate consistency; partial ambiguous dose boundaries
Presence of medication contraindications	44	24	54.55	Moderate consistency; variable difficulty across subcategories
No indication of allergy test and result assessment	2	1	50.00	Low consistency; only one model detected the implicit error
Inappropriate solvent selection	26	11	42.31	Low consistency; requires specialized pharmaceutical compatibility knowledge
Presence of drug interactions	33	12	36.36	Low consistency; depends on multi-drug reasoning depth
Medication inconsistent with diagnosis	41	11	26.83	Lowest consistency; requires clinical diagnostic reasoning
**Total**	**270**	**139**	**51**.**48**	—

The bold values were for visual emphasis only and carry no specific meaning.

Rule-based error categories with explicit, well-defined judgment criteria exhibited the highest inter-model consistency. The inappropriate selection of dosage form and administration route category achieved the highest full agreement rate of 77.78%, followed by presence of duplicate drug administration (66.67%). For these items, errors can be identified through direct textual matching and basic pharmacological knowledge, so performance differences across models were minimal.

In contrast, error categories requiring implicit information extraction and multi-step clinical reasoning showed significantly lower agreement. Medication inconsistent with diagnosis had the lowest full agreement rate of only 26.83%, reflecting large disparities in models' clinical reasoning capabilities and mastery of disease-indication correspondences. Drug interaction identification and solvent compatibility judgment also had low agreement rates (36.36% and 42.31%, respectively), as performance on these items depends heavily on the depth of pharmaceutical knowledge embedded in model training corpora.

## Discussion

### Overall performance of LLMs

This study identified a clear, stable performance ranking among the five general-purpose LLMs for prescription review in the Chinese clinical context: Kimi > DeepSeek > Qwen > Doubao > ChatGPT. It should be noted that this cohort includes only one international model (GPT-4o-mini) alongside four Chinese domestic models; the observed performance advantage of the domestic models tested here should therefore not be generalized to all international large language models.

These observed performance disparities likely stem from a combination of factors including model architecture, medical domain adaptation, knowledge integration, and hallucination mitigation, though these mechanisms are not directly tested in the present study and remain plausible interpretations based on observed performance patterns and existing literature.

Kimi achieved the highest detection rate of 70.37%, the only model exceeding the 70% threshold. It demonstrated well-rounded performance with no obvious weaknesses across question categories or clinical specialties. Plausible explanations for its strong performance include four potential technical strengths. First, robust long-context understanding may support comprehensive retrieval of clinical guidelines, drug labels and literature, enabling accurate parsing of professional terminology and complex logic in prescriptions. Second, strong multi-source information integration and reasoning may allow reliable assessment of complicated prescriptions from neurology and hematology. Third, it may produce far fewer factual errors and can detect subtle specification issues missed by peer models. Fourth, it appears highly adapted to local medical practice; specialized training on Chinese clinical vocabulary and prescription criteria may grant it unique strengths for domestic application.

Ranking second, DeepSeek reached an overall detection rate of 64.81%. It performs strongly on rule-based basic tasks, especially dosage evaluation and routine contraindication checks, and exhibits superior output stability compared with most general-purpose LLMs. However, it appears to struggle with tasks requiring nuanced professional judgment, including solvent selection and drug interaction analysis.

Qwen (56.67%) and Doubao (52.22%) fell into the middle tier. As general-purpose models lacking sufficient medical domain fine-tuning, they are likely hindered by insufficient domain knowledge, limited complex reasoning and frequent medical hallucinations. Still, the two models have respective strengths: Qwen shows notable proficiency in detecting drug interactions, which may imply dedicated optimization for this function. Doubao delivers consistent overall performance and appears to satisfy basic review needs for routine prescriptions in general clinical specialties, without outstanding advantages in any specific assessment category.

ChatGPT recorded the lowest overall detection rate at 45.19%, exposing poor adaptability of this specific international lightweight model to China's medical environment (17). Its main deficiencies likely include insufficient training on Chinese medical corpora, outdated knowledge, inadequate alignment with local regulations and severe medical hallucinations. These shortcomings suggest it cannot meet the practical requirements for clinical prescription screening in this setting.

The performance gap observed for GPT-4o-mini in this Chinese context likely extends beyond language proficiency and knowledge timeliness, rooted in systemic differences in healthcare systems. First, regarding regulatory compliance, China's Standards for Prescription Review in Medical Institutions mandates 12 core review items covering prescription legality, standardization and rationality. These region-specific and dynamically updated regulations may not be sufficiently incorporated into this international model, leading to frequent misjudgments of prescription legality. Second, prescription review standards differ substantially: Chinese guidelines have unique dosage adjustment rules for special populations (children, the elderly) and specific restrictions on antimicrobial and narcotic drugs, inconsistent with international norms. For example, ChatGPT often applies Western pediatric dosage standards to Chinese children, resulting in 31.25% inaccurate dose evaluations in pediatric prescription items. Third, clinical pathway disparities may widen the gap: Chinese hospitals have standardized pathways adapted to local patient characteristics, while this international model was trained primarily on Western cases, leading to poor detection rates in judging medication-diagnosis consistency for regionally prevalent diseases such as viral hepatitis. Finally, this model has limited coverage of Chinese generic drugs, traditional Chinese medicine injections and medical insurance-restricted drugs, failing to identify China-specific irrational medication issues.

It is critical to emphasize that the observed performance advantage of Chinese-developed LLMs should not be interpreted as evidence of inherent superiority in overall model architecture or general reasoning capability. Instead, this gap is plausibly driven by regional training-data bias and differential alignment with local regulatory systems. Chinese models are trained on extensive corpora drawn from China's internet ecosystem, which may give them far deeper exposure to domestic medical terminology, localized clinical practice guidelines, national pharmaceutical regulations, China-specific drug catalogs, and regionally prevalent disease patterns. In contrast, GPT-4o-mini as the single tested international model has more limited access to Chinese-language medical databases, locally restricted regulatory documents, and region-specific clinical resources, placing it at a structural disadvantage in this China-focused evaluation. It is reasonable to hypothesize that other international LLMs would demonstrate comparable or stronger performance when assessed against the clinical standards and training data ecosystems of their home regions. Accordingly, all findings from this study should be interpreted primarily within the Chinese clinical and regulatory context.

From a global regulatory perspective, these findings align with core principles of the US FDA's Artificial Intelligence/Machine Learning (AI/ML)-Based Software as a Medical Device (SaMD) framework and the EMA's regulatory strategy on AI/ML medical devices. Both authorities mandate transparent data provenance documentation and dedicated validation against local formularies, clinical guidelines, and target patient populations prior to clinical deployment. The observed performance divergence between regionally trained models reinforces that general-purpose LLMs require targeted local adaptation and context-specific validation to meet regulatory requirements for safe clinical use across different jurisdictions.

### Performance differences by question type

The eight types of prescription review tasks yielded highly polarized results, reflecting a typical trait of general-purpose LLMs: proficiency in text matching, but weaknesses in professional reasoning and implicit information extraction. We classified all question types into three detection-rate tiers to illustrate the performance limits of the evaluated models.

#### High-detection-rate categories

Tasks concerning inappropriate dosage forms and administration routes fell into this tier. With standardized content and simple textual descriptions, they only require basic text matching instead of sophisticated clinical reasoning. All five models attained detection rates over 65%. Kimi (88.89%) and DeepSeek (83.33%) achieved performance close to dedicated prescription review software. This is the sole task category that general-purpose LLMs can handle dependably.

#### Medium-detection-rate categories

Included here are dosage evaluation, contraindication screening and duplicate drug identification, with overall model detection rate ranging from 50% to 75%. Though guided by well-defined pharmacological rules, these tasks involve basic dose computation, drug component comparison and rule verification. While most models could spot obvious errors, they were prone to miscalculating dosages, neglecting drug components and misapplying relevant rules, leading to poor stability.

#### Low-detection-rate categories

This tier consists of medication-diagnosis alignment, drug interaction analysis, solvent compatibility and allergy test compliance. Detection rate was mostly under 50%, and some models scored below 20% on core items. These tasks depend heavily on professional clinical knowledge, multi-stage reasoning and recognition of implicit information. Evaluating whether medications match diagnoses requires integrated judgment referring to disease guidelines, drug indications and individual patient status. Verifying allergy test compliance means capturing implicit review criteria from prescriptions. All these work demands extensive domain expertise and advanced reasoning skills. In our tests, only Kimi identified a few allergy test-related problems, whereas the rest of the models detected none. This observation supports existing evidence that LLMs are inherently limited in multi-step clinical reasoning and implicit information mining (18, 19).

Notably, the zero-shot prompting protocol employed in this study may contribute to the particularly low detection rate observed in these reasoning-intensive categories. Tasks requiring layered clinical deduction are especially sensitive to prompt structuring, and targeted strategies such as chain-of-thought instructions or few-shot annotated prescription examples could potentially improve detection performance for these complex error types.

### Performance differences by clinical specialty

Noticeable performance stratification was observed across the 10 clinical specialties, which correlated closely with prescription complexity, training corpus coverage and clinical review criteria. This finding reflects the uneven knowledge coverage inherent to general-purpose LLMs.

#### High-detection-rate clinical specialties

Respiratory medicine was the highest-performing clinical specialty with all models attaining detection rates over 65%. Focusing on common illnesses, this clinical specialty adopts standardized medication protocols and presents typical prescription issues. Given its high clinical prevalence, relevant data are sufficiently included in training corpora, resulting in excellent rule matching and model adaptability.

#### Medium-detection-rate clinical specialties

Gastroenterology, neurology and cardiovascular medicine were classified into the medium tier, with model detection rates between 50% and 70%. Clear pharmacological rules apply in these general clinical fields, allowing the models to identify basic prescription errors. Nevertheless, they frequently failed to detect problems associated with complex polypharmacy and individualized medication.

#### Low-detection-rate clinical specialties

Overall detection rates in oncology, pain management, orthopedics and pediatrics was mostly under 50%. ChatGPT achieved scores below 36% across orthopedics, oncology and pediatrics. These disciplines are highly specialized, featuring complex treatment regimens, strict personalized dosing rules and multiple clinical restrictions. Oncology prescriptions covering chemotherapy, targeted therapy and immunotherapy are particularly complicated, making evaluation of drug interactions and contraindications difficult. For pediatric prescriptions, doses must be calculated accurately according to age and weight, complying with strict safety rules for special populations. In addition, existing LLM training datasets are heavily skewed toward medications for common diseases, with limited samples for specialist care and special populations. Such data scarcity causes insufficient professional expertise and impairs the models' capacity to conduct reliable prescription reviews in these clinical specialties.

Notably, these low detection-rate figures are partially attributable to the text-only nature of our prescription vignettes. Real-world clinical prescription validation for pediatric and oncologic populations relies heavily on dynamic patient-specific parameters-including body weight, body surface area, serum creatinine, estimated glomerular filtration rate, liver function tests, and therapeutic drug monitoring levels-that are not embedded in standalone prescription text. This finding underscores that effective clinical deployment of LLM-based prescription review requires integration with electronic health record systems rather than isolated prescription text parsing, to access the full clinical context necessary for accurate, personalized medication assessment.

### Comparison between general-purpose LLMs and professional prescription review software

This study demonstrates that Kimi, the best-performing LLM with a detection rate of 70.37%, still presents a substantial performance deficit relative to commercial professional systems for rational drug use. The discrepancies extend beyond computational efficiency to fundamental distinctions in technical framework, knowledge architecture and clinical applicability.

Widely used professional prescription review software in China is built upon authoritative pharmaceutical repositories, clinical guidelines and drug monographs. Equipped with structured, traceable and dynamically updated rule engines, these systems attain an overall performance exceeding 95% and deliver comprehensive medication risk screening, alongside seamless interoperability with hospital information systems (20, 21).

Conversely, the five general-purpose LLMs evaluated herein had an omission rate of up to 29.63% for the leading model and over 50% for the lowest-performing one-figures that fail to meet the minimum safety standards for clinical prescription review. The key gaps are elaborated below:

#### Knowledge architecture

Professional software relies solely on national formularies, clinical medication guidelines, official drug labels and evidence-based principles as the gold standard for review. All embedded rules are validated by pharmacy experts to ensure authority and rigor (22–24). General-purpose LLMs are pre-trained on open web datasets, resulting in heterogeneous medical knowledge with poor traceability and frequent interference from non-specialized content. Lacking standardized pharmacological rule sets, they are prone to factual inaccuracies and flawed rule-based judgments (25–27).

#### Clinical data integration

Professional systems can retrieve real-time structured patient data covering age, hepatic and renal function, allergy records, medication history and laboratory findings to enable personalized, precise prescription evaluation (28). LLMs are restricted to parsing standalone prescription text and cannot interface with hospital clinical information platforms, resulting in incomplete patient context. This accounts for their unsatisfactory performance in individualized reviews, such as dose titration for special populations and contraindication evaluation for organ impairment, and explains their low detection rates in pediatrics and hematology (29, 30).

#### Rule maintenance and regulatory compliance

Dedicated pharmacy teams continuously update the rule libraries of professional software to incorporate new drugs, revised guidelines and medical insurance policies, keeping review criteria aligned with evolving clinical practice (31, 32). LLMs, however, require complete retraining for knowledge iteration, which causes significant temporal lag. They insufficiently cover newly approved drugs and updated specifications, and adapt poorly to local pharmaceutical regulations in China, leading to non-compliance judgments (33).

#### Explainability and clinical accountability

All outputs from professional software can be traced to specific pharmacological rules and literature references, enabling full traceability, re-audit and clear responsibility allocation. As black-box systems, LLMs have non-transparent reasoning workflows, and their review outcomes lack solid evidence support. Ambiguous responsibility attribution for medication errors induced by model outputs poses considerable clinical and regulatory risks (34, 35).

### Clinical application positioning of general-purpose LLMs

Considering the inherent limitations of general-purpose LLMs and strict clinical compliance rules, such models are not qualified to perform standalone prescription review. Human-AI collaboration currently represents the only safe, compliant and viable approach for real-world clinical application (36).

In this mode, general-purpose LLMs act solely as preliminary screening aids. They efficiently identify straightforward rule violations related to dosage forms, administration routes and standard dosing regimens, and highlight apparent errors. As a front-line filter, they effectively alleviate pharmacists' workload, while being excluded from the review of complex and high-risk prescriptions and barred from making final clinical judgments. Pharmacists retain full authority over prescription review: they validate model outputs, catch sophisticated medication problems and niche risks overlooked or misinterpreted by LLMs, and bear ultimate accountability for medication safety.

However, it is critical to recognize that AI-generated outputs cannot exist in isolation-they must be embedded within structured, pharmacist-led clinical service frameworks to deliver meaningful clinical impact. Automated AI alerts require pharmacist verification and should trigger formalized clinical activities including comprehensive medication review, patient-tailored medication education, and interprofessional consultation with prescribing physicians. Recent real-world implementations demonstrate that well-structured clinical pharmacy services improve medication safety and patient outcomes through systematic medication management and targeted patient engagement (37, 38). Without integration into such established clinical workflows, AI screening tools risk generating disconnected, unactionable alerts or creating redundant workflow inefficiencies. Thus, the true clinical value of LLMs in prescription review will be realized not through standalone operation, but through their role as a front-end catalyst within a well-defined, pharmacist-driven clinical service ecosystem that ensures appropriate follow-up, patient communication, and interprofessional care coordination.

Three fundamental compliance principles must be enforced for human-AI collaborative workflows:

#### Data governance

Deploy models locally within hospital systems and fully de-identify patient information to safeguard privacy.

#### Application governance

All model outputs require manual verification by pharmacists prior to clinical use. It is forbidden to take model results directly as clinical decision evidence.

#### Accountability governance

Pharmacists shall be established as the main responsible entity for prescription review. The auxiliary nature of LLMs and the scope of accountability for clinical practitioners need to be clearly defined.

Future work should focus on targeted professional enhancement, such as refining pharmaceutical knowledge repositories, optimizing model architectures and tailoring functions for clinical scenarios. These improvements will mitigate the deficiencies of LLMs in pharmacy practice and maximize their clinical utility in prescription screening.

### Limitations

First, a unified prompt was used across all prescriptions without task-specific optimization. While this ensured strict experimental consistency, it may have hindered the performance of models on complex reasoning tasks such as multi-drug interaction detection. Future work should explore tailored prompt engineering strategies for different prescription error types.

Second, potential data contamination cannot be completely excluded. Although our test items from the Chinese Pharmacist Skills Competition have not been publicly released, LLMs are trained on massive web corpora that inevitably contain pharmaceutical textbooks, clinical guidelines, and pharmacy examination-style content. Minor content overlap may have slightly inflated the observed performance; however, the consistent superiority of Chinese models across all task categories strongly suggests that contamination alone cannot explain the clear performance ranking observed in this study.

Third, our evaluation was confined to text-only prescription data. In real-world clinical practice, pharmacists routinely integrate multi-source patient information-including laboratory test results, complete medication histories, organ function assessments, and imaging reports-to conduct comprehensive prescription reviews, none of which were included in our test dataset. Our findings may therefore overestimate the practical utility of LLMs in complex, information-rich clinical environments.

Fourth, the gold standard for evaluation was established exclusively based on Chinese clinical guidelines, national formularies, and pharmaceutical regulations, which limits the cross-national generalizability of our results. Model performance may differ substantially when tested against the prescribing standards, drug catalogs, and regulatory frameworks of other countries.

Fifth, false-positive findings were not systematically assessed. Because the gold standard consisted of predefined medication problems, additional incorrect warnings generated by the models were recorded qualitatively but were not included in the primary outcome. The reported proportions therefore represent detection rates (sensitivity) rather than overall accuracy. Future studies should construct a comprehensive reference standard containing both positive and negative items to evaluate false positives, specificity, and clinical utility.

## Conclusion

Based on standardized test items retrieved from the Chinese Pharmacist Skills Competition question bank, this study compared the prescription review performance of five mainstream large language models. The results revealed distinct performance stratification across the included models: Kimi yielded the highest detection rate, followed sequentially by DeepSeek. Overall, domestic Chinese LLMs outperformed ChatGPT. All models achieved satisfactory detection rates in identifying rule-based prescription errors concerning inappropriate dosage forms and administration routes. Nevertheless, they showed notable deficiencies when processing complex prescriptions from specialized clinical fields such as pediatrics and oncology, accompanied by generally high omission rates. These performance differences may be related to variations in training data, domain adaptation, and model design, but further research is needed to confirm the underlying mechanisms. At present, general-purpose LLMs are incapable of replacing professional dedicated prescription review software or manual pharmacist review. Instead, they can only serve as auxiliary tools for preliminary prescription screening. Future research should focus on optimizing LLMs by supplementing professional pharmaceutical knowledge bases and enhancing their clinical logical reasoning capacity to improve their prescription review performance.

## Data Availability

The original contributions presented in the study are included in the article/Supplementary Material, further inquiries can be directed to the corresponding author/s.
